# Dynamic Metabolic Rewiring Enables Efficient Acetyl Coenzyme A Assimilation in Paracoccus denitrificans

**DOI:** 10.1128/mBio.00805-19

**Published:** 2019-07-09

**Authors:** Katharina Kremer, Muriel C. F. van Teeseling, Lennart Schada von Borzyskowski, Iria Bernhardsgrütter, Rob J. M. van Spanning, Andrew J. Gates, Mitja N. P. Remus-Emsermann, Martin Thanbichler, Tobias J. Erb

**Affiliations:** a Max Planck Institute for Terrestrial Microbiology, Marburg, Germany; b Faculty of Biology, Philipps-Universität, Marburg, Germany; c Department of Molecular Cell Biology, Vrije Universiteit, HV, Amsterdam, The Netherlands; d School of Biological Sciences, University of East Anglia, Norwich, United Kingdom; e School of Biological Sciences, University of Canterbury, Christchurch, New Zealand; f Biomolecular Interaction Centre, University of Canterbury, Christchurch, New Zealand; g LOEWE Center for Synthetic Microbiology, Marburg, Germany; University of Washington

**Keywords:** *Paracoccus denitrificans*, acetate, carbon metabolism, ecophysiology, metabolic regulation

## Abstract

During growth, microorganisms have to balance metabolic flux between energy and biosynthesis. One of the key intermediates in central carbon metabolism is acetyl coenzyme A (acetyl-CoA), which can be either oxidized in the citric acid cycle or assimilated into biomass through dedicated pathways. Two acetyl-CoA assimilation strategies in bacteria have been described so far, the ethylmalonyl-CoA pathway (EMCP) and the glyoxylate cycle (GC). Here, we show that Paracoccus denitrificans uses both strategies for acetyl-CoA assimilation during different growth stages, revealing an unexpected metabolic complexity in the organism’s central carbon metabolism. The EMCP is constitutively expressed on various substrates and leads to high biomass yields on substrates requiring acetyl-CoA assimilation, such as acetate, while the GC is specifically induced on these substrates, enabling high growth rates. Even though each acetyl-CoA assimilation strategy alone confers a distinct growth advantage, P. denitrificans recruits both to adapt to changing environmental conditions, such as a switch from succinate to acetate. Time-resolved single-cell experiments show that during this switch, expression of the EMCP and GC is highly coordinated, indicating fine-tuned genetic programming. The dynamic metabolic rewiring of acetyl-CoA assimilation is an evolutionary innovation by P. denitrificans that allows this organism to respond in a highly flexible manner to changes in the nature and availability of the carbon source to meet the physiological needs of the cell, representing a new phenomenon in central carbon metabolism.

## Abstract (precis)

Central carbon metabolism provides organisms with energy and cellular building blocks during growth and is considered the invariable “operating system” of the cell. Here, we describe a new phenomenon in bacterial central carbon metabolism. In contrast to many other bacteria that employ only one pathway for the conversion of the central metabolite acetyl-CoA, Paracoccus denitrificans possesses two different acetyl-CoA assimilation pathways. These two pathways are dynamically recruited during different stages of growth, which allows P. denitrificans to achieve both high biomass yield and high growth rates under changing environmental conditions. Overall, this dynamic rewiring of central carbon metabolism in P. denitrificans represents a new strategy compared to those of other organisms employing only one acetyl-CoA assimilation pathway.

## INTRODUCTION

During growth, bacteria have to distribute metabolic flux between catabolism and anabolism. An important central metabolic pathway is the tricarboxylic acid (TCA) cycle, which interfaces anabolism and catabolism. In the tricarboxylic acid (TCA) cycle, the C_2_-unit acetyl coenzyme A (acetyl-CoA) is catabolized into CO_2_, generating reducing equivalents and ATP for the cell. At the same time, the TCA cycle produces several intermediates that are committed to biosynthesis.

Note that the TCA cycle poses a special challenge for many compounds that are exclusively metabolized via acetyl-CoA, such as acetate, alcohols, short- and long-chain fatty acids, esters, and waxes. Because all carbon of acetyl-CoA is lost to CO_2_, this allows energy conservation but not carbon assimilation through the TCA cycle. Consequently, dedicated pathways for the assimilation of acetyl-CoA are required to allow growth on these ubiquitous substrates.

Two different acetyl-CoA assimilation pathways in bacteria have been described: the glyoxylate cycle (GC) (1, 2) and the ethylmalonyl CoA pathway (EMCP) (3, 4). The GC uses two enzymes in addition to the enzymes of the TCA cycle. The first enzyme of the GC, isocitrate lyase (Icl), cleaves isocitrate into succinate and glyoxylate. This step is followed by the condensation of glyoxylate with a second molecule of acetyl-CoA to form malate and free CoA in a reaction catalyzed by the second enzyme of the GC, malate synthase (1, 2).

The EMCP also forms malate and succinate. However, unlike the GC, the EMCP is a linear pathway that employs 13 different enzymes that collectively convert a total of three acetyl-CoA and two CO_2_ molecules into the TCA cycle intermediates malate and succinate. The key enzyme of the EMCP is crotonyl-CoA carboxylase/reductase (Ccr), which catalyzes the reductive carboxylation of crotonyl-CoA to ethylmalonyl-CoA (3–5).

**FIG 1:**
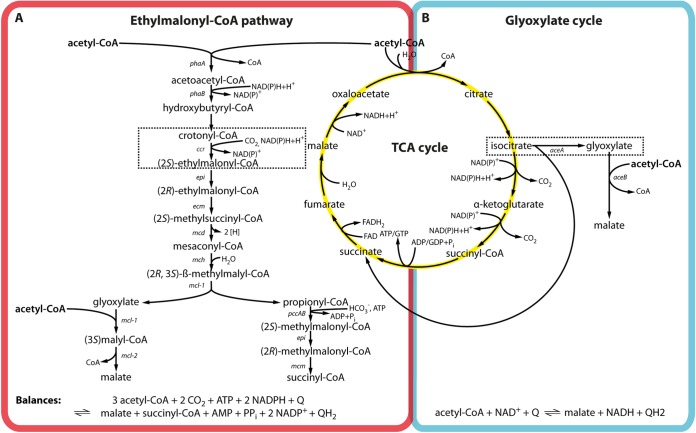
Fate of acetyl-CoA in anabolism and catabolism. Acetyl-CoA is either oxidized to CO_2_ in the tricarboxylic acid (TCA) cycle to generate reducing equivalents and ATP/GTP or assimilated into biomass. Two acetyl-CoA assimilation pathways are present in bacteria, the ethylmalonyl-CoA pathway (A) and the glyoxylate cycle (B). Reaction balances of the individual pathways are shown on the bottom. Note that the activation of acetate to acetyl-CoA requires the hydrolysis of one ATP into AMP and PP_i_ (not included in balance). Key reactions of the individual pathways are indicated in dashed boxes. Genes encoding the individual enzymes of the EMCP and GC are named according to the nomenclature established for Rhodobacter sphaeroides 2.4.1 and Escherichia coli K-12, respectively. The corresponding enzyme names and open reading frames in *P. denitrificans* are listed in Table 1.

Overall, the GC and the EMCP differ substantially in terms of cofactor and carbon requirements. The GC requires four ATPs and generates reducing equivalents at the level of two nicotinamides as well as two quinols per four acetates converted into malate (estimated change in reaction Gibbs free energy [Δ*_r_G*′°] = −129 kJ/mol). To generate two malates from acetate in the EMCP, three molecules of acetate and two CO_2_ molecules are converted at the expense of three ATPs and two reduced nicotinamides, while two quinols are generated (estimated Δ*_r_G*′° = −142 kJ/mol). When acetate is used to generate the CO_2_ and fuel the cycle, this changes the stoichiometry to three consumed ATPs and one reduced nicotinamide, as well as three quinols, generated (estimated Δ*_r_G*′° = −190 kJ/mol). While the EMCP requires more enzymatic steps (and likely more cellular resources), it enables the fixation of inorganic carbon into biomass, as well as the coassimilation of different C-5 and C-3 carbonic acids compared to in the GC, indicating that the two pathways provide different physiological advantages. In most bacteria, only one of the two acetyl-CoA assimilation pathways is present. Importantly, the alphaproteobacterium Paracoccus denitrificans encodes the enzymes for both acetyl-CoA assimilation pathways (Fig. 1), raising the question of which role each individual pathway plays in the cell.

In this work, we show that P. denitrificans uses both the GC and the EMCP. The EMCP serves as the default acetyl-CoA assimilation pathway, which is always present with low activity in the cell during growth on various substrates, including carbon sources that do not require acetyl-CoA assimilation. In contrast, the GC is specifically induced after an organism switches to carbon sources that depend on acetyl-CoA assimilation, such as acetate or crotonate. We further demonstrate that the two acetyl-CoA assimilation pathways confer distinct physiological advantages. Growth with the EMCP results in increased biomass yield in P. denitrificans, while the GC allows for higher growth rates, suggestive for a rate yield tradeoff between the two pathways. Phylogenetic analysis indicates that P. denitrificans and several other alphaproteobacteria acquired the GC through lateral gene transfer, consisting with a specific adaptation of the central carbon metabolism of these organisms during evolution. Collectively, our experiments show a surprising flexibility in central carbon metabolism of alphaproteobacteria that allows the complete rewiring of metabolic flux depending on the type and availability of the carbon and free-energy source according to the physiological needs of the cell.

## RESULTS

### *P. denitrificans* uses the EMCP and the GC for acetyl-CoA assimilation.

Earlier studies suggested that *Paracoccus* might use the EMCP as well as the GC for acetyl-CoA assimilation (4, 6, 7). We therefore searched the genome of the fully sequenced strain *P. denitrificans* Pd1222 for homologs of genes involved in the EMCP and the GC from Rhodobacter sphaeroides 2.4.1 and Escherichia coli K-12, respectively. Our analysis verified that *P. denitrificans* has the genetic potential for both the GC and the EMCP (Fig. 1; Table 1) (4).

**TABLE 1:** Genes coding for enzymes of the EMCP and the GC in Rhodobacter sphaeroides 2.4.1 and Escherichia coli K-12, respectively, and their homologues identified in P. denitrificans Pd1222^*a*^

Pathway or cycle	Enzyme	Gene name	Gene ID in:		
			R. sphaeroides 2.4.1	E. coli K-12	P. denitrificans Pd1222
EMCP	β-Kethothiolase	*phaA*	RSP_0745	–	Pden_2026
	Acetoacetyl-CoA reductase	*phaB*	RSP_0747	–	Pden_2027
	**Crotonyl-CoA carboxylase/reductase**	***ccr***	**RSP_0960**	**–**	**Pden_3873**
	Ethylmalonyl-CoA/methylmalonyl-CoA epimerase	*epi*	RSP_0812	–	Pden_2178
	Ethylmalonyl-CoA mutase	*ecm*	RSP_0961	–	Pden_3875
	(*2S*)-Methylsuccinyl–CoA dehydrogenase	*mcd*	RSP_1679	–	Pden_2840
	Mesaconyl-CoA hydratase	*mch*	RSP_0973	–	Pden_0566
	β-Methylmalyl–CoA/l-malyl–CoA lyase	*mcl-1*	RSP_1771	–	Pden_0799
	(*S*)-Malyl–CoA thioesterase	*mcl-2*	RSP_0970	–	Pden_0563
	Propionyl-CoA carboxylase	*pccAB*	RSP_2191/2189	–	Pden_3684/3688
	Ethylmalonyl-CoA/methylmalonyl-CoA epimerase	*epi*	RSP_0812	–	Pden_2178
	(*2R*)-Methylmalonyl–CoA mutase	*mcm*	RSP_2912	–	Pden_3681

GC	**Isocitrate lyase**	***aceA***	**–**	**C5Y66_09370**	**Pden_1363**
	Malate synthase	*aceB*	–	C5Y66_09365	Pden_1364

a Key enzymes of the two pathways are in bold. Missing enzyme homologues are indicated by dashes.

**FIG 2:**
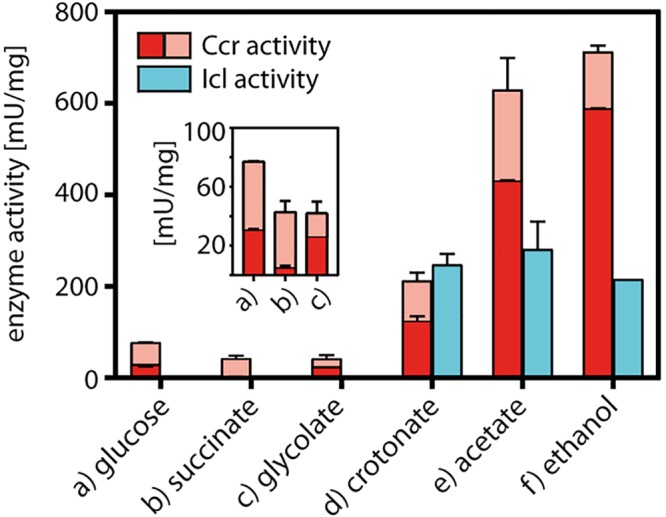
Ccr and Icl enzyme activities in cell extracts of P. denitrificans grown on different carbon sources. Ccr activity is given as a combined activity of reduction and reductive carboxylation of crotonyl-CoA, quantified in the absence (light red) and presence (dark red) of NaHCO_3_. Error bars indicate standard deviations.

To test whether both the EMCP and the GC are functional and thus operate in *P. denitrificans*, we grew bacterial cells in batch culture on minimal medium supplemented with different carbon sources and quantified the activity of Ccr, the key enzyme of the EMCP, as well as Icl, the key enzyme of the GC, in cell extracts obtained from the cultures at mid-log phase (Fig. 2).

The activity of Ccr was almost undetectable in cells grown on succinate and at a very low basal level (<100 mU/mg) in extracts of cells grown on glucose and glycolate. In contrast, the activity of Ccr was significantly higher in extracts of cells grown on crotonate, acetate, and ethanol. In these extracts, Icl activity was also present, while no Icl activity was detected in extracts of cells grown on glucose, succinate, or glycolate. Together, our data suggest that both the EMCP and the GC are active in *P. denitrificans*. However, while the EMCP is always active at low levels and upregulated in response to growth on substrates requiring acetyl-CoA assimilation, the GC is specifically activated only during the growth of *P. denitrificans* on these substrates.

### The EMCP is sufficient to sustain the growth of *P. denitrificans* on acetate.

Next, we tested whether both pathways are essential for acetyl-CoA assimilation in *P. denitrificans*. To that end, we aimed to selectively block the GC or EMCP using external inhibitors. Several inhibitors of Icl are known (8). We screened three of them to identify 3-nitropropionate (3-NPA [9]) as a potential candidate for a selective GC inhibitor of *P. denitrificans in vivo*.

**FIG 3:**
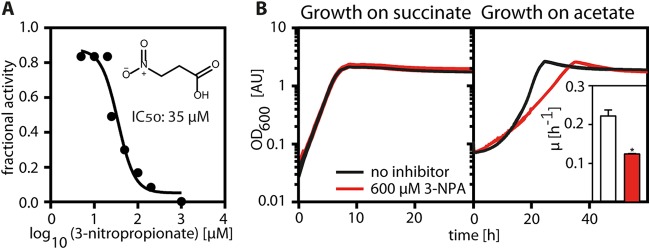
Inhibition of P. denitrificans Icl by 3-NPA *in vitro* (A) and *in vivo* (B). (A) Recombinant His_6_-tagged Icl was preincubated at 30°C for 10 min with increasing concentrations of 3-NPA before activity measurements. The fractional activity of Icl is plotted against the log_10_ of the 3-NPA concentration. The apparent IC_50_ was determined using the log(inhibitor) versus response function of GraphPad Prism7. (B) Growth of P. denitrificans on succinate and acetate in the absence (black line) and presence (red line) of 600 μM 3-NPA. The growth rates on acetate are shown in the inset. Growth on acetate is significantly inhibited by 3-NPA (*P* < 0.5 as determined by an unpaired t test using GraphPad Prism7). Error bars indicate standard deviations.

3-NPA inhibited purified *P. denitrificans* Icl *in vitro* with an apparent 50% inhibitory concentration (IC_50_) of 34 μM at concentrations of 100 μM d,l-isocitrate (Fig. 3A). Moreover, 3-NPA did not affect the growth of *P. denitrificans* on carbon substrates that do not require Icl activity, such as succinate (Fig. 2B; see also Fig. S1E in the supplemental material). Notably, 3-NPA also did not affect the growth of Methylobacterium extorquens AM1, an EMCP-positive organism that is closely related to *P. denitrificans*, on acetate (Fig. S1B and E). This suggested to us that 3-NPA acts as a GC inhibitor that shows negligible off-target effects in the cell, although the possibility of additional effects of 3-NPA could not be completely excluded.

10.1128/mBio.00805-19.1 FIG S1 Testing 3-NPA as a suitable compound for the inhibition of Icl in P. denitrificans *in vivo*. 3-NPA was added to acetate minimal medium inoculated with E. coli DH5α (A), M. extorquens AM1 (B), M. extorquens AM1 lacking *ccr* but heterologously expressing the glyoxylate shunt of E. coli, and P. denitrificans DSM413 (D). (E) Growth rates corresponding to the growth curves in panel B. Despite an extension of the lag phase, the growth rate of M. extorquens AM1 was not affected by the presence of 3-NPA. n.s., not significant. (F) Growth curves of P. denitrificans DSM413 on substrates which do not require acetyl-CoA assimilation, in the absence and presence of 3-NPA, with the corresponding growth rates. Error bars indicate standard deviations. Download FIG S1, TIF file, 2.2 MB. Copyright © 2019 Kremer et al. 2019 Kremer et al. This content is distributed under the terms of the Creative Commons Attribution 4.0 International license.

When we added increasing concentrations of 3-NPA to *P. denitrificans* growing on acetate, the growth rate was successively decreased from 0.26 ± 0.043 h^−1^ (0 μM 3-NPA) to 0.13 ± 0.005 h^−1^ (600 μM 3-NPA) but not completely abolished (Fig. 3B; Fig. S1D). This indicated that the GC is not essential in *P. denitrificans* and that the EMCP is sufficient for acetyl-CoA assimilation.

### The use of the GC increases the growth rate of *P. denitrificans* on acetate.

**FIG 4:**
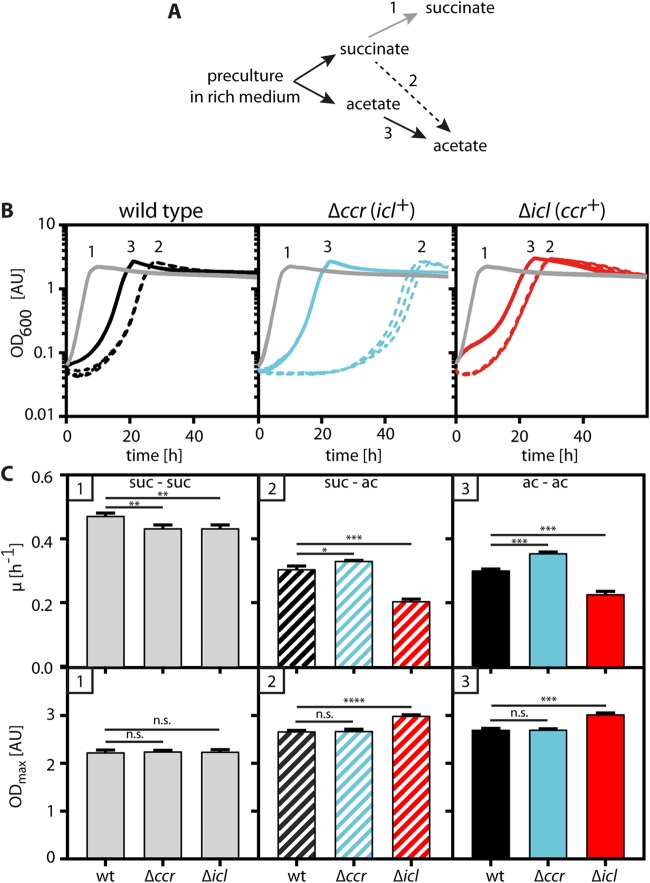
Phenotypic characterization of the Δ*ccr* and Δ*icl* strains. (A) Inoculation scheme. (B) Growth behavior of wild-type, Δ*ccr*, and Δ*icl* P. denitrificans strains on succinate (gray lines; 1), after a switch from succinate to acetate (dashed lines; 2), or from acetate to acetate (solid lines; 3). The curves show independently grown triplicates, and in some cases the lines overlap. (C) Growth rates and maximum optical densities calculated from the curves shown in panel B. Error bars indicate standard deviations. Asterisks mark the level of significance of the growth rate or maximal optical density between the wild type (wt) and the individual mutants as determined by *t* test, with the number of asterisks indicating the decimal place of *P* (e.g., ***, *P* < 5 × 10^−3^; n.s., not significant). AU, arbitrary units; suc, succinate; ac, acetate.

To estimate the contribution of each pathway to acetyl-CoA assimilation, we individually deleted the genes coding for Ccr and Icl from the genome of *P. denitrificans*, yielding Δ*ccr* and Δ*icl* strains, respectively (Fig. 4; Fig. S2). During growth on succinate, both deletion strains grew similarly to the wild type. However, a shift from succinate to acetate caused pronounced and different strain-specific growth defects.

10.1128/mBio.00805-19.2 FIG S2 Enzyme activities in *ccr* and *icl* deletion strains. To confirm the absence of Ccr and Icl in the respective single-deletion backgrounds, enzyme activities were determined in cell extracts of the deletion strains grown to mid-exponential phase in acetate minimal medium. n.d., not detectable. Dark red, Ccr activity with concomitant carboxylation; light red, Ccr activity independent of carboxylation. Error bars indicate standard deviations. Download FIG S2, TIF file, 1.0 MB. Copyright © 2019 Kremer et al. 2019 Kremer et al. This content is distributed under the terms of the Creative Commons Attribution 4.0 International license.

The Δ*ccr* strain grew with wild type-like growth rates but displayed an extended lag phase (Fig. 4). Notably, this lag phase ranged from 15 to 50 h between different experiments (Fig. S3). However, when the Δ*ccr* strain was subcultured on acetate, the variation in the lag phase disappeared (Fig. 4B, solid blue line), and the growth rate corresponded to that of the wild type (Fig. 4B and C), indicating that the mutant adapted to acetate.

10.1128/mBio.00805-19.3 FIG S3 Growth of individual clones of the P. denitrificans Δ*ccr* strain on acetate and succinate. Cells were grown in rich medium, washed, and shifted to either acetate or succinate minimal medium. While all clones exhibited the same growth behavior on succinate, they displayed different lag phases during growth on acetate. The genotypes of all clones were confirmed by colony PCR after completion of the experiment. Download FIG S3, TIF file, 1.4 MB. Copyright © 2019 Kremer et al. 2019 Kremer et al. This content is distributed under the terms of the Creative Commons Attribution 4.0 International license.

The Δ*icl* strain, in contrast, did not exhibit a prolonged lag phase but showed a decreased growth rate on acetate, which is consistent with the results of the 3-NPA inhibition experiments. Together, these results suggest that the GC enables fast growth of *P. denitrificans* on acetate, but unlike with the EMCP, some time is required for the strain’s growth to be induced after a switch to acetate.

### The EMCP and GC show a complex expression pattern during switches from succinate to acetate.

**FIG 5:**
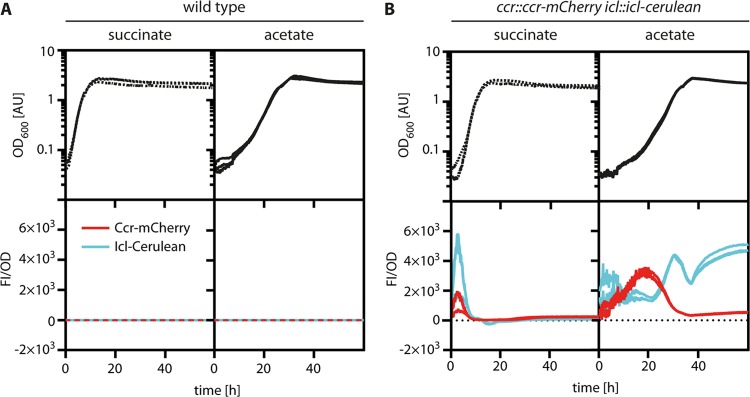
Expression of Ccr and Icl in P. denitrificans during growth on succinate and acetate. The optical density (OD_600_) and fluorescence intensity (FI) of the P. denitrificans wild type (A) and *ccr*:*ccr*-mCherry *icl*::*icl*-Cerulean strain (B) were monitored during growth on succinate and acetate using a 96-well plate reader. Fluorescence was normalized to the OD and corrected by the background signal of the wild type. The Ccr-mCherry level is shown in red, and the Icl-Cerulean level is shown in cyan. Growth curves and fluorescent measurements from triplicates are shown, and in some cases the lines overlap.

To follow the expression of the EMCP and the GC *in vivo*, the native *ccr* and *icl* genes of *P. denitrificans* were replaced with constructs encoding translational fusions of Ccr to the red fluorescent protein mCherry and of Icl to the cyan fluorescent protein Cerulean, respectively. The resulting strain (*P. denitrificans ccr*::*ccr-*mCherry *icl*::*icl-*Cerulean) was continuously monitored by determining the total fluorescence of the population during cultivation in 96-well plates (Fig. 5).

During growth on succinate, cells showed very low levels of mCherry fluorescence and even lower levels of Cerulean fluorescence, which is consistent with our earlier finding that basal Ccr, but no Icl, activity could be detected in cell extracts of *P. denitrificans* grown on succinate (Fig. 2). When shifted from succinate to acetate, the reporter strain showed biphasic *ccr-*mCherry and *icl-*Cerulean expression patterns. Ccr-mCherry fluorescence increased within the first 20 h and then decreased gradually at the onset of exponential phase until it again reached a low basal level. In contrast, the production of Icl-Cerulean started only after 20 h but continued to increase until shortly before the cells entered stationary phase. Subsequently, Icl-Cerulean fluorescence dropped transiently before continuing to rise again in the late stationary phase. Very similar growth-linked expression patterns were observed in reporter strains of *P. denitrificans* carrying only a single Ccr-mCherry or Icl-mCherry fusion (Fig. S5). In summary, our results suggest that acetyl-CoA assimilation in *P. denitrificans* follows a complex regulatory pattern in which the GC is used for fast and efficient acetyl-CoA assimilation during exponential phase, while the EMCP is used to bypass the time needed for activation of the GC.

10.1128/mBio.00805-19.5 FIG S5 Expression of Ccr-mCherry and Icl-mCherry in P. denitrificans single-reporter strains during growth on succinate and acetate. Succinate and acetate cultures were started from washed succinate-grown precultures. Growth curves and fluorescent measurements from triplicates are shown, and in some cases, the lines overlap. Fluorescence measurements of the individual strains were performed with different gain adjustments. Therefore, the absolute fluorescence intensity values cannot be compared between the different strains. Download FIG S5, TIF file, 2.6 MB. Copyright © 2019 Kremer et al. 2019 Kremer et al. This content is distributed under the terms of the Creative Commons Attribution 4.0 International license.

### Population-wide switch responses monitored by single-cell microscopy.

**FIG 6:**
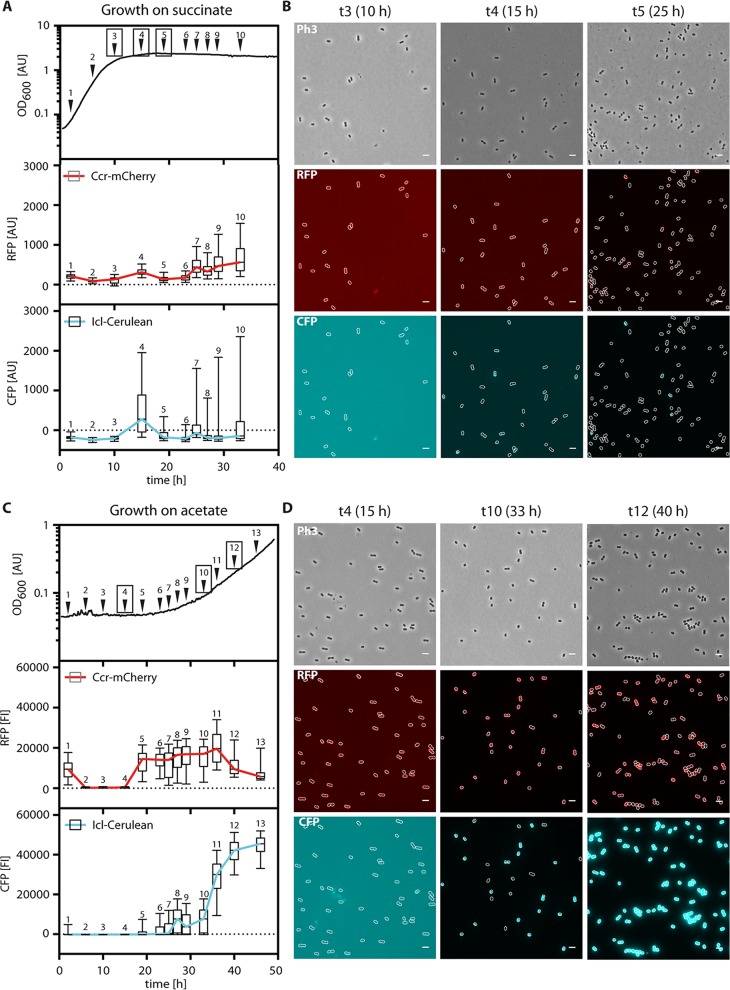
Fluorescence of P. denitrificans *ccr*::*ccr*-mCherry *icl*::*icl*-Cerulean during growth on succinate (A, B) and acetate (C, D). At the indicated time points, the cultures were sampled and analyzed microscopically. Images were subjected to automated analysis using BacStalk (10). (A and C) Distribution of cellular fluorescence intensities (*n* = 450 cells per time point) shown as box plots. The values were corrected for the background fluorescence of the wild type. The margins of the boxes encompass the 25th to 75th percentiles, the lines indicate the median, and the whiskers extend to the 5th and 95th percentiles, respectively. The colored lines connect the medians. (B and D) Representative microscopy images. Ph3, phase contrast; RFP, red fluorescent protein, mCherry channel (overlaid with Ph3); CFP, cyan fluorescent protein; Cerulean channel (overlaid with Ph3).

To understand whether the transition from EMCP- to GC-driven acetyl-CoA assimilation during the switch to acetate occurred in the whole population or in only a subset of cells, we followed the production of Ccr-mCherry and Icl-Cerulean in *P. denitrificans ccr*::*ccr-*mCherry *icl*::*icl-*Cerulean at the single-cell level using time-lapse fluorescence microscopy. Here, we imaged cells growing on succinate (Fig. 6A and B) and acetate (Fig. 6C and D) at 2-h intervals and subjected the images to automated analysis (10) to determine the average fluorescence per cell (Fig. 6A and C). While repeated handling of the cultures for sample collection led to slightly decreased growth rates, the overall expression patterns of *ccr-*mCherry and *icl-*Cerulean matched those measured during continuous cultivation in the 96-well plates (Fig. 5).

While no fluorescence was detected for the wild type (negative control) (Fig. S4), the reporter strain showed different production patterns for Ccr-mCherry and Icl-Cerulean. On succinate, Ccr was produced at low levels during exponential phase, with increased expression in the stationary phase. In contrast, Icl was detected only at the end of the exponential phase and only in a small fraction of cells, which appeared strongly fluorescent. This implies that Icl expression is heterogeneous in the late exponential phase on succinate and thus may suggest a bet-hedging-like behavior under these conditions (Fig. 6A and B; Fig. S6).

10.1128/mBio.00805-19.4 FIG S4 Fluorescence of the P. denitrificans wild type. Cells were grown on succinate and on acetate and analyzed microscopically to exclude the possibility that fluorescence detected in the analysis of P. denitrificans *ccr*::*ccr*-mCherry *icl*::*icl*-Cerulean stemmed from autofluorescence of P. denitrificans. Scale bar, 3 μm. Download FIG S4, TIF file, 2.0 MB. Copyright © 2019 Kremer et al. 2019 Kremer et al. This content is distributed under the terms of the Creative Commons Attribution 4.0 International license.

10.1128/mBio.00805-19.6 FIG S6 Comparison of Ccr-mCherry (RFP) to Icl-Cerulean (CFP) fluorescence. RFP and CFP intensities of the P. denitrificans *ccr*::*ccr*-mCherry *icl*::*icl*-Cerulean strain grown on succinate and acetate (growth and individual fluorescence intensities are shown in Fig. 6) are plotted as logarithmic ratios. Gray points represent the values of individual cells. The red lines mark the medians of the sampled populations. Download FIG S6, TIF file, 2.1 MB. Copyright © 2019 Kremer et al. 2019 Kremer et al. This content is distributed under the terms of the Creative Commons Attribution 4.0 International license.

Upon the switch to acetate, Ccr was produced first, whereas Icl was induced only at the onset of the early exponential phase. While some cells switched earlier to Icl production than others, all cells produced Icl in the exponential phase, indicating that the whole population and not only a subset of cells shifted from using the EMCP to using the GC (Fig. 6C and D; Fig. S6).

**FIG 7:**
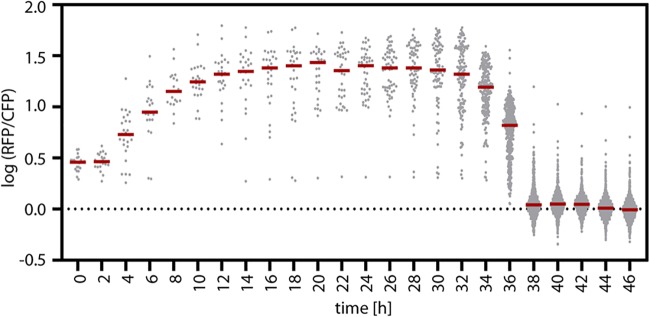
Comparison of Ccr-mCherry (RFP) to Icl-Cerulean (CFP) fluorescence in single cells over time. Succinate-grown cells of the strain P. denitrificans *ccr*::*ccr*-mCherry *icl*::*icl*-Cerulean were immobilized in a microfluidic system with a continuous flow of acetate minimal medium. Cell growth and fluorescence intensities were tracked by time-lapse fluorescence microscopy, with observation of the same field of view for 46 h. The fluorescence intensities of individual cells, determined via automated image analysis (38), are plotted as the logarithmic ratio of the RFP and CFP intensities. Gray points represent the values of individual cells. The red lines mark the medians of the sampled populations.

To follow the expression dynamics of *ccr* and *icl* continuously in individual cells, we trapped succinate-grown *P. denitrificans ccr*::*ccr-*mCherry *icl*::*icl-*Cerulean in a microfluidic device. We then provided acetate as the sole source of carbon and tracked cell growth and division as well as reporter production by microscopy over the course of time (Movie S1). This analysis confirmed that essentially all cells switched from the EMCP to the GC, demonstrating that faster acetyl-CoA assimilation is switched on in the whole population and not by individual cells that outgrow the population (Fig. 7).

10.1128/mBio.00805-19.9 MOVIE S1 Fluorescence of P. denitrificans *ccr*::*ccr*-mCherry *icl*::*icl*-Cerulean during growth on acetate after a switch from succinate. Cells were grown in succinate, trapped in a microfluidic device, and continuously flushed with acetate minimal medium. Images were taken every 2 h; time points are indicated on the individual frames of the video. Scale bar, 3 μm. Download Movie S1, AVI file, 3.4 MB. Copyright © 2019 Kremer et al. 2019 Kremer et al. This content is distributed under the terms of the Creative Commons Attribution 4.0 International license.

## DISCUSSION

Here, we describe an unprecedented (and unexpected) metabolic redundancy in bacterial central carbon metabolism. The chromosome of *P. denitrificans* carries genes for two fundamentally different acetyl-CoA assimilation pathways, the EMCP and the GC. Both pathways are functional and regulated dynamically depending on the growth stage of the cells. The EMCP serves a default function and is expressed at basal levels at all times and on all carbon and free-energy sources tested. Upon a switch to acetate, the EMCP is strongly induced at the early stage of growth but then decreases in activity again. The GC, in contrast, is exclusively induced on acetate and reaches peak activity at the late stage of growth, indicating a surprising rewiring of central carbon metabolism in *P. denitrificans* at the onset of the exponential phase.

What might be the reasons for the rewiring of central carbon metabolism in *P. denitrificans*? Individual knockout strains of the EMCP and GC show that the two pathways confer distinct advantages. The EMCP increases the growth yield, while the GC allows faster growth on acetate. How can these differences be explained, and what are their consequences?

Importantly, the EMCP is able to fix inorganic carbon to increase biomass yield according to the following equation: 3 acetate + 2 CO_2_ + 2 NADPH + 2 quinones → 2 malate + 2 NADP^+^ + 2 quinols. This stoichiometry allows organisms using the EMCP to gain extra carbon from the environment if additional reducing equivalents are available to the cell, e.g., through internal storage compounds, such as polyhydroxybutyrate (PHB), or through growth substrates that are comparatively more reduced than average cellular carbon. This is supported by recent calculations that predict an approximately 3%-higher yield for M. extorquens on methanol when the EMCP is used than when the GC is used (11). Another important feature of the EMCP is that it not only enables acetyl-CoA assimilation but also is directly linked to PHB metabolism and can also function in the assimilation of propionate, in addition to several dicarboxylic acids. This versatility makes the EMCP an all-purpose pathway that might allow *P. denitrificans* to assimilate several different carbon and free-energy sources in parallel, which may explain the constant expression of the EMCP on diverse growth substrates.

The GC, on the other hand, is a very specialized route that requires only two additional enzymes. The GC requires fewer protein resources, and the thermodynamics of the cycle (i.e., the free energy of the overall process) might become more favorable with the upper limit of the free Gibbs energy dissipation rate in *P. denitrificans* (12). Rerouting acetyl-CoA assimilation to the GC thus might allow a higher metabolic flux and consequently faster growth, providing a potential explanation as to why *P. denitrificans* switches to the GC when high concentrations of acetate are available.

Overall, a picture emerges in which metabolic rewiring is a highly coordinated strategy in *P. denitrificans* that allows optimal growth of this species in a changing environment. When the growth substrate changes from succinate to acetate, it is conceivable that the constant expression of the EMCP facilitates the immediate assimilation of acetate. This might help *P. denitrificans* during the lag phase until the GC is fully induced and thus provide an advantage over other microorganisms that rely on the GC only. Once induced, the GC then may enable increased acetate assimilation rates, which might enable *P. denitrificans* to outcompete microorganisms that possess solely the EMCP.

**FIG 8:**
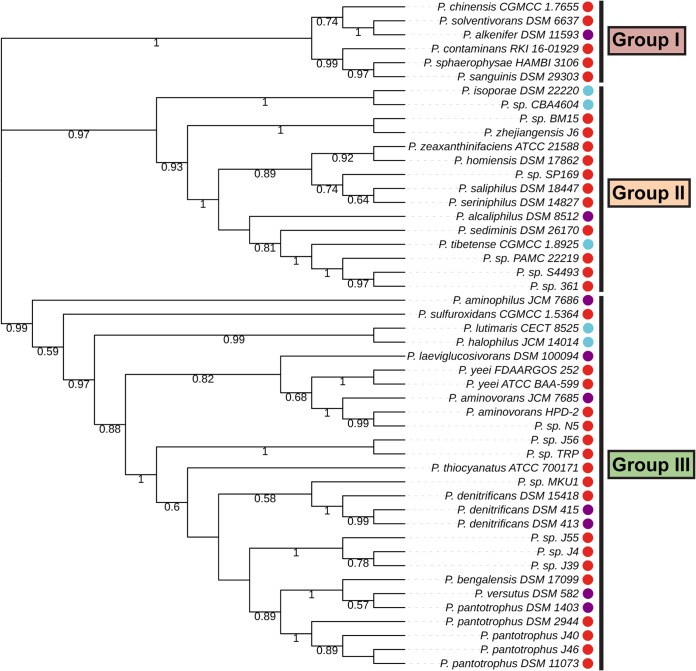
Maximum-likelihood phylogenetic tree of *Paracoccus* strains. The phylogenetic tree is based on the concatenated alignments of 12 protein sequences from 48 *Paracoccus* strains. Bootstrap values of ≥0.5 are given on the respective nodes; calculated branch lengths of the tree are ignored for the sake of easier visualization. The presence of Ccr is marked with a red dot, the presence of Icl is marked with a blue dot, and the presence of both enzymes is marked with a purple dot. The 48 strains are clustered in three distinct groups.

Out of 48 *Paracoccus* genomes analyzed, 34 possess only a *ccr* homolog, while 9 additionally contain a homolog of *icl* (Fig. 8; see also Fig. S7 and S8 in the supplemental material). Only five strains seem to exclusively possess an *icl* homolog. Notably, *ccr* phylogeny correspond largely to overall strain phylogeny, suggesting that the EMCP is the ancient acetyl-CoA assimilation strategy in the genus *Paracoccus*. This hypothesis is in line with our experimental observation that the EMCP serves a default function in *P. denitrificans* and is further supported by the fact that other, closely related alphaproteobacteria possess only the EMCP (4).

10.1128/mBio.00805-19.7 FIG S7 Maximum-likelihood phylogenetic tree of Ccr sequences. The phylogenetic tree is based on Ccr sequences from 43 *Paracoccus* strains. Bootstrap values of ≥0.5 are given on the respective nodes; calculated branch lengths of the tree are ignored for the sake of easier visualization. The presence of Ccr is marked with a red dot, and the presence of both Ccr and Icl is marked with a purple dot. The clustering of the 43 strains is largely similar to the phylogeny shown in Fig. 8. Download FIG S7, TIF file, 2.6 MB. Copyright © 2019 Kremer et al. 2019 Kremer et al. This content is distributed under the terms of the Creative Commons Attribution 4.0 International license.

10.1128/mBio.00805-19.8 FIG S8 Maximum-likelihood phylogenetic tree of Icl sequences. The phylogenetic tree is based on Icl sequences from 14 *Paracoccus* strains. Bootstrap values of ≥0.5 are given on the respective nodes; calculated branch lengths of the tree are ignored for the sake of easier visualization. The presence of Icl is marked with a blue dot, and the presence of both Ccr and Icl is marked with a purple dot. Note that the Icl-positive strains of *Paracoccus* can be found in all three phylogenetic groups of the genus (compare Fig. 8). Download FIG S8, TIF file, 1.8 MB. Copyright © 2019 Kremer et al. 2019 Kremer et al. This content is distributed under the terms of the Creative Commons Attribution 4.0 International license.

Notably, the phenomenon of metabolic rewiring is presumably not restricted to the *Paracoccus* clade alone. Several alphaproteobacteria show the genetic potential to express multiple acetyl-CoA assimilation pathways (4), suggesting that dynamic rewiring of the central carbon metabolism is a more widespread strategy in nature. This discovery extends recent findings that metabolic degeneracy plays an important role in alphaproteobacterial metabolism (13). Future experiments will focus on understanding the molecular mechanisms that underlie the coordination of the two acetyl-CoA pathways and their regulation through transcriptional, posttranslational, or allosteric regulatory mechanisms (14–17) and on clarifying the evolutionary and ecological significance of dynamic metabolic rewiring as a new principle in microbial central carbon metabolism.

## MATERIALS AND METHODS

### Strains, media, and growth conditions.

All experiments were performed with Paracoccus denitrificans strains DSM413 and Pd1222 (Table 2). The strain preferentially used for all experiments was DSM413, which is the original wild-type strain isolated by Beijerinck and Minkman in 1908 (18). In cases where genetic manipulation of this strain was not successful, its genetically more tractable derivative Pd1222 (19) was used alternatively. *P. denitrificans* was grown at 30°C in mineral salt medium (trace elements, TE3-Zn) (20) supplemented with defined carbon sources and adjusted to a total carbon concentration of 120 mM. The density of cultures was determined photometrically at a wavelength of 600 nm. Bacterial growth was monitored over time using Tecan Infinite 200 Pro plate reader systems (Tecan Trading AG, Switzerland) with Nunclon Delta Surface 96-well plates (Thermo Scientific, USA). *In silico* analyses of growth data were performed using the software Prism 7 (GraphPad Software, USA). Escherichia coli was grown at 37°C in Luria-Bertani broth or M9 minimal medium (337 mM NaH_2_PO_4_, 220 mM KH_2_PO_4_, 85.5 mM NaCl, 18.7 mM NH_4_Cl, 1 mM MgSO_4_, 0.3 mM CaCl_2_, 0.13 mM Na_2_EDTA, 0.03 mM FeSO_4_, 1 μg/ml biotin, 1 μg/ml thiamine) supplemented with 60 mM acetate. Methylobacterium extorquens AM1 was grown as described previously (21) at 30°C in mineral medium supplemented with 10 mM acetate. Antibiotic concentrations were used as follows: kanamycin at 25 or 50 μg ml^−1^, spectinomycin at 50 μg ml^−1^, and tetracycline at 10 μg ml^−1^.

**TABLE 2:** Strains used in this study

Strain	Genotype or relevant feature	Source or reference
E. coli TOP 10	Cloning strain	Invitrogen, USA
E. coli DH5α	Cloning strain	ThermoFisher Scientific, USA
E. coli BL21	Protein expression	ThermoFisher Scientific, USA
E. coli ST18	Mating strain	28
M. extorquens AM1	Wild type	39
P. denitrificans DSM413	Wild type	18
P. denitrificans Pd1222	Increased conjugation frequency	19
P. denitrificans DSM413 Δ*ccr* (TJE-KK5)	Δ*ccr*	This work
P. denitrificans DSM413 Δ*icl* (TJE-KK6)	Δ*icl*	This work
P. denitrificans Pd1222 *ccr*::*ccr-*mCherry *icl*::*icl-*Cerulean (TJE-KK14)	*ccr*::*ccr-*mCherry *icl*::*icl-*Cerulean	This work
P. denitrificans Pd1222 *ccr*::*ccr-*mCherry (TJE-KK13)	*ccr*::*ccr-*mCherry	This work
P. denitrificans Pd1222 *icl*::*icl-*mCherry (TJE-KK10)	*icl*::*icl-*mCherry	This work

### Chemicals.

All chemicals were obtained from Sigma-Aldrich (Steinheim, Germany) and Carl Roth (Karlsruhe, Germany) and were of the highest purity available.

### Construction of plasmids.

Oligonucleotides and synthetic genes were purchased from Eurofins Deutschland (Hamburg, Germany). All standard cloning techniques were carried out according to the instructions in reference 22.

Markerless genomic deletions and integrations of reporter genes were generated using the pK18*mobsacB* sucrose suicide plasmid system (23).

For the deletion of *ccr* (*Pden_3873*) and *icl* (*Pden_1363*), respectively, fusions of the respective downstream and upstream flanking regions of the genes, each approximately 700 bp in length, were purchased as synthetic genes cloned into the pEX-K4 backbone (pSYNccrflanks [pTE1602] pSYNiclflanks [pTE1604]) (Table 3). Restriction of the plasmids with PstI and EcoRI, gel purification of the flanking regions, and subsequent ligation into PstI/EcoRI-digested pK18*mobsacB* resulted in plasmids pK18*mobsacB*_ccrflanks (pTE1606) and pK18*mobsacB*_iclflanks (pTE1615).

**TABLE 3:** Oligonucleotides used for plasmid construction and mutant verification in this study

Oligonucleotide	Sequence	Purpose
NdeI_pKK21-f	TATATCATATGCCCCGGCCCCGGCGCATG	Generation of pTE1624
pKK21-r	TATATCCTAGGGCGGCGCCCCGCCTCCAGCGCGTC	
mCherry-f	TATATCCTAGGGCAGGGAGTGCGGCCGGCAG	
NdeI_mCherry-r	TATATCATATGTCACTTGTACAGCTCGTCCATG	

NdeI_pSYNiclXiclds-f	TATACATATGTCTAGAGGGCCGACAGGATTCGGCC	Generation of pTE1625
HindIII_pSYNccrxccrds-r	TATATAAGCTTGCCGCTGCCGGCCGCACTCCCTGC	
HindIII_cerulean-f	TATATAAGCTTATGGTGAGCAAGGGCGAGGAGC	
NdeI_cerulean-r	TATATCATATGTTACTTGTACAGCTCGTCCATGCCGAG	

NdeI_icl-f	TATACATATGAGCAGAAAGACTTTTTCGGAAATC	Generation of pTE1614
HindIII_icl-r	TATAAAGCTTCTATTCGGCGGCGAACTGGTTCATGGTG	

Pden_3873_ds-f	GGTCAGGCGCTTGTATTGGCCGAACATGTAG	Verification of TJE-KK5
Pden_3873_ups-r	GCGAAAGCGGCATCGCCGTGGTGCGGATGAATTAC	

Pden_1363_ds-f2	CATCCATTCATAGGCGGTGACCACCAGGCCC	Verification of TJE-KK6
Pden_1363_ups-r	GCTGGGACTATATCTTCAGCTATATCAAGAC	

Pden_1363_ds-f2	CATCCATTCATAGGCGGTGACCACCAGGCCC	Verification of TJE-KK10
iclmCherryiclds_seq-f	CAGCATTGCCGAGGCCGACTACCCGGAC	
Pden_1363_ds-f	GACGAGCGCCGTGGTGAGTCTCAGCATGATGG	
Pden_1363_ups-r	GCTGGGACTATATCTTCAGCTATATCAAGAC	

NdeI mCherry-r	TATATCATATGTCACTTGTACAGCTCGTCCATG	Verification of TJE-KK13
Pden_3873_ups-r	GCGAAAGCGGCATCGCCGTGGTGCGGATGAATTAC	
3'-ccr-f	CGCAGGCGCATCTGAAGATGC	
Pden_3873_ds-f	GGTCAGGCGCTTGTATTGGCCGAACATGTAG	

Pden_1363_ds-f2	CATCCATTCATAGGCGGTGACCACCAGGCCC	Verification of TJE-KK14
Pden_1363_ups-r	GCTGGGACTATATCTTCAGCTATATCAAGAC	
HindIII_cerulean-f	TATATAAGCTTATGGTGAGCAAGGGCGAGGAGC	

For the introduction of fluorescent reporter fusions, *ccr* and *icl* were ordered as synthetic genes fused to evoglowPp1 (24) and mCherry (25), respectively, each preceded by a 30-bp linker and followed by the downstream region of the respective genes, yielding plasmids pEX4_ccrevoglowPp1ccrds (pTE1601) and pEX4_iclmCherryiclds (pTE1603).

pTE1603 was digested with SalI and BamHI. Subsequent ligation into SalI/BamHI-cut pK18mobsacB yielded pK18*mobsacB*_iclmCherryiclds (pTE1616).

pTE1601 was amplified with targeted omission of the evoglowPp1 gene via inverted PCR using primers NdeI_pKK21-f and pKK21-r. The mCherry gene was amplified from pTE1603 using primers mCherry-f and NdeI_mCherry-r. Restriction of both fragments with AvrII and NdeI and subsequent ligation of the individual fragments to each other resulted in the plasmid pEX4_ccrmCherryccrds. pEX4-ccrmCherryccrds was digested with SalI and BamHI. Subsequent ligation into SalI/BamHI-cut pK18mobsacB yielded pK18*mobsacB*_ccrmCherryccrds (pTE1624).

pTE1603 was amplified with targeted omission of the mCherry gene via inverted PCR using primers NdeI_pSYNiclXiclds-f and HindIII_pSYNccrXccrds-r. The Cerulean gene (26) was amplified from plasmid pVCERC-6 (27) using primers HindIII_cerulean-f and NdeI_cerulean-r. Restriction of both fragments with HindIII and NdeI and subsequent ligation of the individual fragments to each other resulted in the plasmid pEX4_iclceruleaniclds. Restriction of pEX4_iclceruleaniclds with SalI and BamHI and ligation into SalI/BamHI-cut pK18*mobsacB* yielded pK18*mobsacB*_iclceruleaniclds (pTE1625).

For the heterologous expression of *icl*, *icl* was amplified from pTE1603 using primers NdeI_icl-for and HindIII_icl. The resulting product was digested with NdeI and HindIII and ligated into NdeI/HindIII-digested pET16b, yielding pET16b_icl (pTE1614).

### Genetic manipulation of *P. denitrificans*.

The transfer of plasmids into *P. denitrificans* was achieved by biparental mating with the donor strain E. coli ST18 (28). Selection of the first integration was performed on LB or methanol mineral medium plates supplemented with 25 μg/ml kanamycin. Colonies were picked and restreaked on LB with kanamycin and LB with 3% sucrose in parallel. Colonies that were kanamycin resistant and sucrose sensitive were grown in plain LB for 2 days. Subsequently, cells were plated on methanol mineral medium plates supplemented with 6% sucrose in serial dilution. Colonies were restreaked on LB with kanamycin and LB with 3% sucrose in parallel. Kanamycin-sensitive, sucrose-resistant clones were screened for the successful deletion or integration of genes by colony PCR.

### Preparation of cell extracts.

*P. denitrificans* cultures were grown at 30°C in mineral medium supplemented with various carbon sources. Cells were harvested at mid-exponential phase, resuspended in ice-cold morpholinepropanesulfonic acid (MOPS)-KOH buffer (100 mM, pH 7.2), and lysed by sonication (MS 72 microtip, 40%, 15 pulses three times). Cell debris was removed by centrifugation at 35,000 × *g* and 4°C for 1 h. The total protein concentration of the cell extracts was determined with the Bradford assay (29) using bovine serum albumin (BSA) as the standard. The catalytic activities of crotonyl-CoA carboxylase/reductase and isocitrate lyase were measured spectrophotometrically as described previously (3, 30).

### Synthesis of crotonyl-CoA.

Crotonyl coenzyme A was synthesized from its anhydride as described before (31).

### Live-cell imaging.

To monitor gene expression *in vivo*, cells were immobilized on 1% agarose pads and analyzed microscopically using an Axio Observer.Z1 (Zeiss) microscope equipped with a Plan Apochromat 100×/1.4 oil Ph3 phase-contrast objective, an ET-mCherry filter set (Chroma, USA), and a pco-edge sCMOS camera (PCO AG, Germany). Images were recorded with VisiView 3.3.0.6 (Visitron Systems, Puchheim, Germany) and processed with Fiji (32) and Adobe IllustratorCS5 (Adobe Systems, USA). For time-lapse imaging, cells were transferred to a B04 CellASIC ONIX2 microfluidic plate (Merck, Germany) coupled to an ONIX EV262 microfluidic platform (CellASIC, USA), cultivated at 30°C under continuous medium flow (1 lb/in^2^) and imaged at regular intervals using the microscope setup described above. For automated data analysis, images were processed with Fiji (32) and BacStalk (10).

### Heterologous production and purification of 6×His-Icl.

Competent E. coli BL21 cells were transformed with the expression plasmid pET16b_icl and grown at 37°C in terrific broth (23.6 g/liter yeast extract, 11.8 g/liter tryptone, 9.4 g/liter K_2_HPO_4_, 2.2 g/liter KH_2_PO_4_, and 4% [vol/vol] glycerol) supplemented with ampicillin. Gene expression was induced at an optical density at 600 nm (OD_600_) of 0.8 by addition of 0.5 mM isopropyl thiogalactopyranoside (IPTG). After additional growth at 18°C for 12 h, cells were harvested, resuspended in 3 volumes of buffer A (500 mM NaCl, 50 mM Tris-HCl, pH 7.9) containing 0.1 mg of DNase I and protease inhibitor cocktail (Sigma-Aldrich, St. Louis, MO, USA), and lyzed by sonication. Lysates were cleared by centrifugation at 42,000 × *g* and 4°C for 45 min. The supernatant was applied onto a preequilibrated 1-ml Ni-Sepharose fast-flow column (HiTrap, FF; GE Life Sciences, United Kingdom) and washed with buffer A. Proteins were eluted from the column by the addition of increasing concentrations of buffer B (500 mM NaCl, 50 mM Tris-HCl, 500 mM imidazole, pH 7.9), followed by application onto a 5-ml HiTrap desalting column (GE Life Sciences, United Kingdom) for desalting and buffer exchange to storage buffer (buffer A, 10% glycerol). Protein concentration was determined using a NanoDrop 2000 spectrometer (Thermo Scientific, USA), and purity was analyzed by SDS-PAGE according to the procedures in reference 33.

### Measurement of enzyme activities in cell extracts and purified protein. (i) Ccr.

The enzyme activity of crotonyl-CoA carboxylase/reductase was measured by monitoring the consumption of NADPH spectrophotometrically as described previously (3).

**(ii) Icl.** The enzyme activity of isocitrate lyase was measured by monitoring the production of a phenylhydrazine-glyoxylate complex as described previously (30). For the Icl inhibition assay with 3-nitropropionate (3-NPA), the compound was preequilibrated in 100 mM MOPS-KOH, pH 7.2, overnight. Purified isocitrate lyase was incubated in the reaction mixture containing 3-NPA at various concentrations at 30°C for 10 min before the assay was started by the addition of 2 mM d,l-isocitric acid.

### Phylogenetic analysis.

Sequences of 12 proteins (GapA, GyrA, RecA, RpoA, RpoB, TrpB, 30S ribosomal protein S2, 30S ribosomal protein S3, 30S ribosomal protein S12, 50S ribosomal protein L2, 50S ribosomal protein L3, 50S ribosomal protein L11) from the core proteome of 48 strains of *Paracoccus* were downloaded from the IMG database (34) and aligned using MUSCLE 3.8.31 (35). The alignments were concatenated and used to calculate a phylogenetic tree with MEGA 7.0.14 (36) using the maximum-likelihood method with the Le-Gascuel substitution model and 100 bootstrap replicates. The resulting tree was visualized using iTOL (37).

Sequences of Ccr and Icl from 43 and 14 strains of *Paracoccus*, respectively, were downloaded from the IMG database and used to calculate maximum-likelihood phylogenetic trees as described above.
